# Gene signature‐based prediction of triple‐negative breast cancer patient response to Neoadjuvant chemotherapy

**DOI:** 10.1002/cam4.3284

**Published:** 2020-07-21

**Authors:** Yanding Zhao, Evelien Schaafsma, Chao Cheng

**Affiliations:** ^1^ Department of Molecular and Systems Biology The Geisel School of Medicine at Dartmouth College Lebanon NH USA; ^2^ Department of Biomedical Data Science The Geisel School of Medicine at Dartmouth College Lebanon NH USA; ^3^ Department of Medicine Baylor College of Medicine Houston TX USA; ^4^ The Institute for Clinical and Translational Research Baylor College of Medicine Houston TX USA

**Keywords:** biomarker, estrogen receptor, gene expression profile, neoadjuvant chemotherapy, triple‐negative breast cancer, tumor microenvironment

## Abstract

Neoadjuvant chemotherapy is the current standard of care for large, advanced, and/or inoperable tumors, including triple‐negative breast cancer. Although the clinical benefits of neoadjuvant chemotherapy have been illustrated through numerous clinical trials, more than half of the patients do not experience therapeutic benefit and needlessly suffer from side effects. Currently, no clinically applicable biomarkers are available for predicting neoadjuvant chemotherapy response in triple‐negative breast cancer; the discovery of such a predictive biomarker or marker profile is an unmet need. In this study, we introduce a generic computational framework to calculate a response‐probability score (RPS), based on patient transcriptomic profiles, to predict their response to neoadjuvant chemotherapy. We first validated this framework in ER‐positive breast cancer patients and showed that it predicted neoadjuvant chemotherapy response with equal performance to several clinically used gene signatures, including Oncotype DX and MammaPrint. Then, we applied this framework to triple‐negative breast cancer data and, for each patient, we calculated a response probability score (TNBC‐RPS). Our results indicate that the TNBC‐RPS achieved the highest accuracy for predicting neoadjuvant chemotherapy response compared to previously proposed 143 gene signatures. When combined with additional clinical factors, the TNBC‐RPS achieved a high prediction accuracy for triple‐negative breast cancer patients, which was comparable to the prediction accuracy of Oncotype DX and MammaPrint in ER‐positive patients. In conclusion, the TNBC‐RPS accurately predicts neoadjuvant chemotherapy response in triple‐negative breast cancer patients and has the potential to be clinically used to aid physicians in stratifying patients for more effective neoadjuvant chemotherapy.

## Graphical abstract

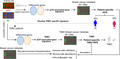

## 1 INTRODUCTION

Neo‐adjuvant chemotherapy (NCT) is the standard‐of‐care for breast cancer and can improve treatment options for patients with large, inoperable, or advanced tumors.^1^ Multiple clinical trials have illustrated the clinical benefits of NCT; in large or inoperable tumors, it has been shown to significantly reduce tumor size and enable conservative breast surgery for certain patients. The survival benefit of NCT is identical to adjuvant chemotherapy (the traditional option) in early‐stage breast cancer patients.^2^, ^3^, ^4^, ^5^ However, in contrast with adjuvant chemotherapy, which is given in the absence of any measurable parameter for response evaluation, the response to NCT is often evaluated by MRI or PET/CT and can be classified as a pathologic complete response (pCR). Of note, patients with pCR have prolonged survival compared to patients with residual disease.^2^, ^6^, ^7^ Despite its clinical advantages, however, only 30% of patients responded to NCT; the majority presents with residual disease (RD) and suffers from side effects that can hamper further surgical options.

To address this issue, a number of genetic signatures have been proposed to predict patient response to NCT and inform treatment decision.^8^, ^9^, ^10^, ^11^, ^12^, ^13^ In ER‐positive breast cancer patients, several cell cycle pathway associated gene signatures have been commercialized due to their high accuracy in predicting response to NCT. One of the most widely utilized signatures is Oncotype DX, which predicts NCT response in ER‐positive breast cancer patients based on the expression of 21 genes.^14^, ^15^, ^16^, ^17^, ^18^ Patients with high Oncotype DX risk scores are more likely to respond to the NCT.^17^ Other gene assays, such as EndoPredict and PROSIGNA, were also introduced to predict the NCT response in ER‐positive patients.^19^, ^20^ While the success of ER‐positive commercialized gene signatures has been promising, very few predictive gene signatures in ER‐negative patients have been reported.

TNBC is a subset of ER‐negative breast cancer, which accounts for 10‐20% of all breast cancers. TNBC tumors fail to express estrogen receptors (ER), progesterone receptors (PR), and the epidermal growth factor receptor‐2 (Her2).^21^, ^22^ Compared to other subtypes, TNBC is the most aggressive and is characterized by larger tumor size, higher grade, increased number of lymph node metastases at diagnosis, and the worst survival outcomes. Unfortunately, current treatment options for TNBC are very limited.^21^, ^23^, ^24^ Indeed, no targeted therapies for TNBC are available, with the exception of PARP inhibitors in germline *BRCA1/2*‐mutated tumors.^25^ This makes chemotherapy the only treatment option for most TNBC patients. Due to the presence of a high intertumoral heterogeneity, the same NCT regimen may yield diverse responses in different patients.^26^ This presents a need for the identification of predictive biomarkers that can be applied to help tailor care. Previously, Stover *et al* reported that both proliferation and immune‐related gene signatures are associated with response in TNBC patients.^27^, ^28^ Farmer *et al* reported that a stroma‐related gene signature was predictive of NCT response in TNBC patients.^29^ However, compared to the Oncotype DX prediction accuracy in ER‐positive patients, none of these signatures achieve a high prediction accuracy, which limits their potential for clinical utilization. Currently, there is no clinically predictive gene signature for TNBC patients.^27^, ^30^ Therefore, developing clinically applicable biomarkers for TNBC to predict NCT response is critical and would spare nonresponder patients from experiencing severe side effects.

In this study, we propose a computational framework to define a whole‐transcriptome signature to quantify the probability of a patient to respond to NCT. To this end, we utilized pretreated patient gene expression data by comparing the NCT responders vs nonresponders to define the gene signature associated with NCT response. Our rationale is that treatment response in cancer involves complicated cellular and molecular interactions in the tumor environment in which, for example, cell metabolism and cell‐cell interactions are important. While most published signatures focus on a single tumor‐associated pathway, we included all genes to capture the complicated cellular and molecular interactions, which more accurately predicts NCT response. Here, we present NCT response associated gene signatures to calculate response probability scores (RPS) in breast cancer patients. Our results indicate the utility of our computational framework for identifying novel predictive biomarker(s) and have identified a powerful biomarker for NCT response prediction in TNBC.

## 2 MATERIALS AND METHODS

### 2.1 Breast cancer gene expression datasets

The Gene Expression Omnibus (GEO) and MD Anderson Cancer Center public databases were queried for available gene expression datasets using the following search terms: (breast cancer) AND (preoperative chemotherapy OR neoadjuvant chemotherapy). Only microarray datasets generated using Affymetrix (U133 and U133Plus2.0 arrays) and having more than 80 samples were included to limit the cross‐platform variability. Patient samples were excluded if the biopsies were obtained after NCT, if the patient sample did not have ER‐status or Her2‐status information, if pathologic response was not available, or if comparable treatment agents were not found (Figure S1). Duplicate records were removed by careful review of GEO annotations. Based on these criteria, we identified seven datasets. The GEO accession numbers and the dataset downloaded from the MD Anderson Cancer Center public database were: GSE25055, GSE20194, GSE25065, GSE20271, GSE32646, GSE22093, and Hess *et al*,^31^, ^32^, ^33^, ^34^, ^35^, ^36^ with sample sizes of 306, 278, 182, 178, 115, 97, and 129 respectively (Table S1 and Figure S1). In total, 115 patient expression profiles were measured by Affymetrix U133Plus2.0 arrays and 1170 patient expression profiles were measured by Affymetrix U133 array. The gene expression data were downloaded as matrices containing the expression level of all probes and then converted into gene‐level expression. For genes with multiple probesets, the probeset with the largest average expression value across samples was selected to represent the expression of that gene.

GSE25055 was used as training dataset for constructing the RPS and TNBC‐RPS signatures, while the other datasets were used as validation datasets. We constructed a validation metadata dataset by applying quantile normalization to re‐scale the RMA normalized gene expression and then applying the ComBat function (“*sva”* R package)^37^ to remove batch effects (Figure S2).

### 2.2 Define RPS and TNBC‐RPS gene signatures

To capture the transcriptome difference between pCR and RD patients, the RPS gene signature was defined by identifying differentially expressed genes between pCR and RD patients (Table S2). A logistic regression model was constructed for each gene using patient class as the response variable (Y = 1 for pCR patients, and Y = 0 for RD patients).

$$\ln\left(\frac{Y}{1‐Y}\right)=\beta_{0}+\beta_{1}X_{1}.$$

Log2‐transformed gene expression data were included as a predictive variable in the model (X_1_). The coefficients (β_1_‐values) and statistical significance (*P*‐values) for each gene were estimated by applying these models to the training data (GSE25055). Then, given these values (β, *P*) for all genes, the RPS gene signature was constructed by using a pair of weight profiles, w^+^ and w^‐^, that assigned all genes which had two weights in the following way: for gene *i*, $w_{i}^{+}=‐\log\left(p_{i}\right)I\left(\beta_{i}>0\right)$ and $w_{i}^{‐}=‐\log\left(p_{i}\right)I\left(\beta_{i}<0\right)$, where *I* represents the indicator function. Weights were trimmed at 10 to avoid extreme values and transformed into values within [0,1] by subtracting the minimum value and then dividing by the range. If a gene *i* was more significantly up‐regulated in pCR vs RD samples, it received a high weight in the $w_{i}^{+}$ profile and a weight of zero in the $w_{i}^{‐}$ profile. Conversely, a more significantly down‐regulated gene in pCR vs RD samples was assigned a high weight in the $w_{i}^{‐}$ profile and weight of zero in the $w_{i}^{+}$ profile. The TNBC‐RPS gene signature was derived based on the same framework, but the logistic regression model was performed for each gene in TNBC patients only (Table S3).

### 2.3 Calculation of RPS and TNBC‐RPS in pretreated breast cancer samples

Given the expression profiles for a number of breast cancer patients, sample‐specific RPSs were calculated for all samples based on the RPS gene signature. Specifically, a modified version of a statistical method called BASE^38^, ^39^, ^40^, ^41^, ^42^ was applied as follows: first, gene expression profiles were median normalized to relative gene expression for each gene across samples. Second, for each sample, its gene expression profile was sorted in a descending order based on the relative expression to obtain an expression profile ($e_{1},e_{2},\ldots ,e_{g}$), where g was the total number of genes. The skewed distribution of up‐regulated (with large values in $w^{+}$) and down‐regulated (with large values in $w^{‐}$) genes in pCR and RD samples were examined by comparing two cumulative functions, a foreground *f(i)* and a background *b(i):*

$$f\left(i\right)=\frac{\sum_{k=1}^{i}\left|e_{k}w_{k}\right|}{\sum_{k=1}^{g}\left|e_{k}w_{k}\right|},\;1\leq i\leq g,$$

$$b\left(i\right)=\frac{\sum_{k=1}^{i}\left|e_{k}\left(1‐w_{k}\right)\right|}{\sum_{k=1}^{g}\left|e_{k}\left(1‐w_{k}\right)\right|},\;1\leq i\leq g.$$

If genes with large weights in *w* ($w_{i}^{+}$ for up‐regulated genes and $w_{i}^{‐}$ for down‐regulated genes in breast cancer samples) also had high gene expression values in a breast cancer sample expression profile *e*, $f\left(i\right)$ would accumulate more rapidly than $b\left(i\right)$ as i increases. Third, for genes in $w_{i}^{+}$, RPS^+^ was defined as the maximum deviation between the $f\left(i\right)$ and $b\left(i\right)$ and then normalized against null distribution that was generated by 1,000 iterations of a randomized tumor expression profile. The same process was applied for genes in $w_{i}^{‐}$ to generate the RPS^‐^. The final RPS was determined by taking the difference between RPS^+^ and RPS^‐^ (RPS^+^‐RPS^‐^). Using this approach, patients receiving high RPSs had profiles similar to gene expression profiles of patients with known pCR, while patients receiving low RPSs had profiles similar to gene expression profiles of patients with known RD.

For the TNBC‐RPS calculation, the TNBC‐RPS signature was applied in the TNBC patients. Following the method above, the foreground *f(i)* and background *b(i)* functions were used to calculate TNBC‐RPS for each TNBC patient. Specifically, for global prediction power comparison, we calculated RPS and TNBC‐RPS based on the expression of metadata. For individual cohort prediction power comparison, we calculated RPS and TNBC‐RPS based on the original normalized expression data.

### 2.4 Previously defined predictive gene signature calculation

Gene signatures were collected from published studies describing a variety of biological processes implicated in chemosensitivity or resistance. Three categories of gene signatures were collected in our study for comparison: Category 1 (ER‐positive patient): Commercialized gene signatures were used for prediction and comparison. Oncotype DX risk scores,^8^ MammaPrint signature scores,^9^ EndoPredict scores,^43^ Gene76 scores,^44^ Genomic Grade Index (GGI),^45^ and risk of recurrence scores (RORs) ^46^ were calculated using the “oncotypeDX”, “gene70”, “endoPredict”, “gene76”, “ggi”, and “rorS” functions, respectively, from the genefu R package.^47^ Moreover, Stover *et al* ^48^ reported Module scores of MammaPrint and GGI were also included. In addition, Ignatiadis *et al* ^28^ examined and compared the predictive power of a total of 17 signatures. We only chose eight signatures that have been examined to be predictive in ER‐positive patients. The Module score of each signature was calculated as follows:

$$\text{Module}\;\text{score}=\frac{\sum_{i=1}^{n}w_{i}e_{i}}{\sum_{i=1}^{n}\left|w_{i}\right|},$$

where $w_{i}$ referred to the weight of the genes in the module and $e_{i}$ referred to the expression of these genes.

Category 2 (ER‐negative and TNBC patient): The search terms: (predict OR biomarker) AND Breast cancer AND (ER negative OR Triple negative) AND (neoadjuvant OR preoperative chemotherapy) were used to find relevant publications. After excluding publications with no gene expression‐based signature, or which were not validated in at least two independent datasets, 19 gene signatures remained.^28^, ^49^, ^50^, ^51^ The methods of calculating those 19 gene signatures were as follows:

*Signature 1:* Witkiewicz *et al* reported that cell‐cycle‐related genes are important for NCT and used nine genes to quantify the related pathway activity.^49^ The average expression of these nine genes were calculated as the metric for prediction.

*Signature 2*: Turner *et al* presented a Consensus Signature ^50^ that captured the combined effect of immune function, tumor proliferation, and the tumor proliferation regulators. In detail, this signature was composed of the sum of the STAT1 module score (immune function), *TOP2A* (tumor proliferation), and *LAPTM4B* (tumor proliferation regulator) gene expression. The Module score was calculated used the equation above. We then scaled the Module score to have an inter‐quartile range of 1 and a median of 0. The expression level of *TOP2A* and *LAPTM4B* was rescaled by the same method. The final score was calculated as the sum of these three scaled scores.

*Signature 3*: Desmedt *et al* ^51^ combined the modules associated with different tumor microenvironment components for prediction. Module scores of the Immune response, Stromal signature, and TOP2A signature (cell proliferation) were calculated through the equation described above. Specifically, the application of the signature was determined by the Her2 status. In ER‐negative/Her2‐negative patients, the final score was calculated as the sum of the Immune response, Module score, and Stromal Module score. In ER‐negative/Her2‐positive patients, the final score was calculated as the sum of the Immune response, Module score, Stromal Module score, and the TOP2A signature Module score.

*Signatures 4‐13*: Ignatiadis *et al* ^28^ reported 10 of 17 signatures that have been examined to be predictive in ER‐negative patients. Similarly, the Module score of each signature was calculated through the equation above.

*Signatures 14‐17:* MammaPrint scores,^43^ GGI,^45^ MammaPrint Module Score and GGI Module Score calculated above were used for prediction.

*Signature 18*: Juul *et al* ^52^ identified that the mitotic and ceramide modules were associated with the pCR and defined the paclitaxel response metagene score as the difference between mitotic Module score and ceramide Module score.

*Signature 19*: Farmer *et al* ^29^ used the stromal‐cell‐associated signature for prediction, which was calculated as the average gene expression of 48 genes.

*Category 3 (Non‐ER‐status dependent)*: Stover *et al* ^48^ reported and summarized 125 signatures from previous studies for NCT prediction. For each gene signature, its Module score was calculated as the metric for prediction. In summary, a total number of 143 signatures were calculated, as described in the accompanying publications, and were validated in corresponding datasets from the original studies (Table S4‐S5). Specifically, for global prediction power comparison, we calculated 143 signature scores based on the expression of metadata. For individual cohort prediction power comparison, we calculated 143 signature scores based on the original normalized expression data.

### 2.5 NCT response prediction

Patients were predicted to have pCR or RD based on scores derived from the RPS, the TNBC‐RPS, and the other 143 signatures collected from previous publications. For each signature, we ranked patients based on signature scores from low to high. For each patient, a threshold was set, beginning with the lowest score, where patients with a score higher than the threshold were predicted to be pCR and patients below the threshold were predicted to be RD. The sensitivity and specificity were then calculated for each threshold by comparing the predicted pCR to the actual pCR. Prediction accuracy of each signature was represented by calculating the area under the receiver operating characteristics curve (AUC).

To evaluate the performance of each signature in combination with established clinical predictors, a Random Forest model was trained to predict pCR and RD status using the RPS, the TNBC‐RPS, and other signatures as predicting features, integrated with clinical predictors including age, tumor stage, and tumor grade. Random Forest classification was performed in R through the *randomForest* package, while setting the sample size of pCR and RD patients to be equal.^53^ The performance of the model was evaluated by a 10‐fold cross validation, where samples were randomly divided into 10 subgroups, with nine subgroups being used to train the Random Forest model and one subgroup used for NCT response prediction. To make each sample part of the validation set at least once, this process was repeated 10 times. Model prediction accuracy was evaluated by calculating AUC. This overall cross‐validation procedure was repeated 100 times to obtain an overall average AUC.

### 2.6 Pathway enrichment analysis and tumor microenvironment component decomposition

The MsigDB C2 dataset ^54^ was downloaded for pathway enrichment analysis. KEGG gene sets, BioCarta genes sets, and Reactome gene sets were chosen for analysis. Gene sets with less than 20 genes were excluded, which lead to the inclusion of 798 pathways. For each pathway gene set, the enrichment score was calculated based on the rank of pathway genes in the RPS and TNBC‐RPS gene signatures. Specifically, the enrichment score was calculated through a walking sum method:

$$\text{Enrichment}\;\text{score}=\left(\frac{\sum_{i=1}^{n}g_{i}*d_{i}}{n*N}‐0.5\right)*2,$$

where $g_{i}$ referred to the accumulative hits of genes in the gene set, $d_{i}$ referred to the gene rank difference between two continuous hits in the RPS or TNBC‐RPS signatures, *n* referred to the total number of genes in the gene set, and *N* referred to the total number of genes in the RPS or TNBC‐RPS gene signatures.

The tumor microenvironment was decomposed into three general components: infiltrating immune cells, stromal cells, and tumor cells. In detail, the abundance of infiltrating immune cells and stromal cells in the tumor microenvironment were estimated using the *ESTIMATE* package in R.^55^ The proliferation rate of tumor cells was estimated using the normalized expression level of *MKI67*.^56^

## 3 RESULTS

### 3.1 Overview of the study

**FIGURE 1:**
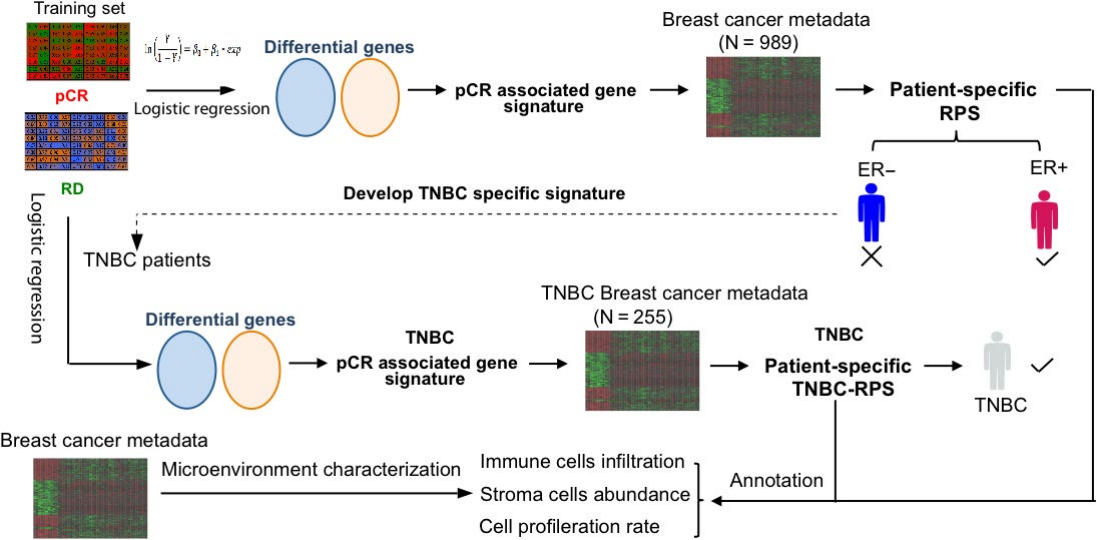
The schematic diagram of this study. GSE25055 microarray data were used to determine the gene signature that captures the gene expression difference between pCR and RD patients. The signature was applied to the breast cancer metadata to calculate the patient‐specific response probability score (RPS). RPS predicts response better in ER‐positive patients than ER‐negative patients. Following this, the TNBC gene signature was defined by using TNBC only in the GSE25055 microarray data. The signature was applied to the TNBC patients in the metadata to calculate the TNBC response probability score (TNBC‐RPS) and was validated in the TNBC. The annotation of the gene signatures was performed by correlating the RPS or TNBC‐RPS with the immune cell, stromal cell abundance, and tumor cell proliferation rate in the tumor microenvironment

We developed a computational framework that could be utilized to identify predictive gene signatures associated with neoadjuvant chemotherapy (NCT) response in triple‐negative breast cancer (TNBC) and then conducted a series of analyses as summarized in Figure 1. We compared pretreatment gene expression profiles between pathologic complete response (pCR) and residual disease (RD) patients from a prospective clinical study (GSE25055) to identify a weighted whole‐gene signature associated with NCT response, where genes are weighted based on their capacity to discriminate pCR vs RD patients. A response probability score (RPS) was calculated for each patient in the metadata (see methods) through a rank‐based algorithm called BASE.^38^ Patients having high similarities between their gene expression profile and NCT response associated signature would have high RPS scores, leading to high probability of being pCR. After illustrating the efficacy of the framework by showing that the RPS has similar predictive power as the leading commercialized signatures, such as Oncotype DX and MammaPrint in ER‐positive patients. We expanded the framework in TNBC patients, generated a novel TNBC response‐associated signature, calculated TNBC response probability scores (TNBC‐RPS) for TNBC patients in the metadata, and examined its predictive power in TNBC. Moreover, we annotated RPS and the TNBC‐RPS by correlating the scores with immune cell infiltration, stromal cell abundance, and tumor cell proliferation rate in the tumor microenvironment.

### 3.2 The RPS predicts patient response with high accuracy in ER‐positive breast cancer

**FIGURE 2:**
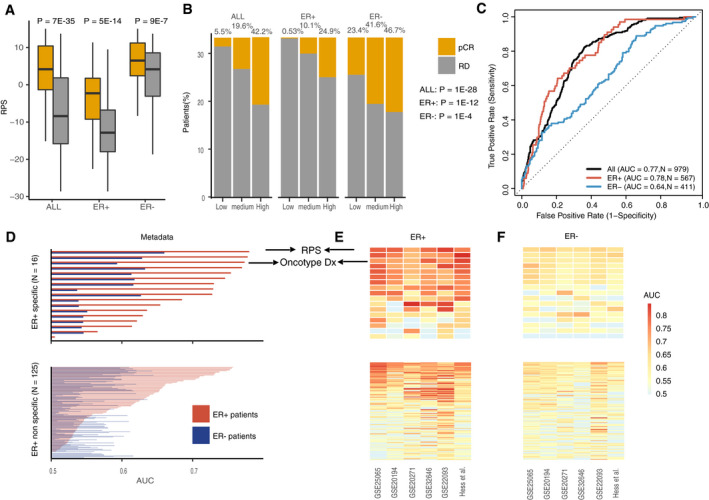
The RPS predicts NAC response better in ER‐positive patients than ER‐negative patients. (A) RPS is higher in pCR than RD samples. All patients, ER‐positive, and ER‐negative patients are labeled as “ALL,” “ER+”, and “ER‐“ respectively. The statistical significance is calculated by Wilcoxon rank sum test; (B) pCR patients are enriched in the high‐RPS group. Patients are separated based on the tertile of their RPS. The pCR patients are significantly enriched in the high‐RPS group. The statistical significance is calculated by Chi‐square test; (C) RPS predicts patients’ response. Receiver Operating Characteristic (ROC) curves for pCR prediction using RPS as feature. ROC curves were generated for all (*black*), ER‐positive (*red*), and ER‐negative (*blue*) patients; (D) Comparison of RPS with public signature prediction power in ER‐positive and ER‐negative patients. Public signatures are separated into ER‐positive‐specific and ‐nonspecific predictive signatures. Barplot shows the area under the curve (AUC) difference between the RPS and other public signatures in ER‐positive and ER‐negative patients; (E) ER‐positive patients in six individual datasets; (F) ER‐negative patients in six individual datasets. Comparison of the RPS with other public signature prediction power in ER‐positive and ‐negative patients across six individual datasets. “Up” panel corresponds to ER‐positive‐specific signatures and “down” panel is corresponding to non‐ER‐positive‐specific signatures

A number of gene signatures have been proposed for ER‐positive breast cancer, including several commercialized assays such as Oncotype DX.^8^, ^9^, ^10^, ^11^, ^12^, ^13^ We first tested our framework in ER‐positive breast cancer by comparing its performance with commercialized assays. The efficacy of our developed computational framework was validated by investigating the predictive power of the RPS for NCT response (Figure S3A‐B and Figure 2). As shown in Figure 2A, patients with pCR had a significantly higher RPS than patients with RD (*P* = 7e‐35, Figure 2A). Because ER‐negative patients had a higher response rate to NCT than ER‐positive patients,^57^ we separated the patients by ER status. In both ER‐positive and ‐negative patients, pCR patients had a significantly higher RPS than RD patients (*P* = 5e‐14, ER‐positive patients; *P* = 9e‐7, ER‐negative patients; Figure 2A). Similar results were observed in the enrichment analysis, that pCR patients were significantly enriched in the high RPS group compared to other groups (*P* = 1e‐28, All patients; *P* = 1e‐12, ER‐positive patients; *P* = 1e‐4, ER‐negative patients Figure 2B). Furthermore, to quantify the predictive power of the RPS, we utilized the RPS as a predictor to classify patients as pCR or RD. As shown in Figure 2C, RPS was predictive to NCT response and Higher predictive power was observed in ER‐positive patients (AUC = 0.77, All patients; AUC = 0.78, ER‐positive patients; AUC = 0.64, ER‐negative patients, Figure 2C and Table S6).

To compare the predictive performance of RPS to the commercialized signatures, we collected 143 predictive signatures in breast cancer from previous publications (see methods). These signatures could be stratified based on their applicable range, including ER‐positive‐specific signatures, ER‐negative‐specific signatures, and nonspecific signatures (see methods). We applied all collected signatures in the metadata to examine and compare their AUC with the RPS in ER‐positive and ‐negative patients. Compared with ER‐positive‐specific predictive signatures, the RPS had similar or higher AUC performance in ER‐positive patients compared to most of the ER‐positive‐specific signatures (Figure 2D and Table S6). Interestingly, MammaPrint and Oncotype DX had an AUC of 0.78 and 0.77 in ER‐positive patients, respectively, while the RPS had an AUC of 0.78 in ER‐positive patients (Figure 2D and Table S6). For convenience, we grouped ER‐negative‐specific and nonspecific signatures together and named this group “ER‐positive‐nonspecific signatures”. In these nonspecific signatures, the loss of *RB1* expression, a cell proliferation signature, and the epithelial‐mesenchymal transition (EMT) signature had the highest AUC of 0.76 in ER‐positive patients. Notably, no robust signatures were identified in ER‐negative patients (Figure 2D and Table S6).

To show that the predictive accuracy of RPS in the metadata was not driven by a single dataset, we then examined and compared the predictive consistency of RPS with 143 other signatures in ER‐positive patients from each dataset. As shown in Figure 2E, the RPS was predictive of the response in each individual dataset, with the lowest AUC = 0.71 in the GSE20271 dataset. This was similar to other commercially available predictive signatures, including Oncotype DX (lowest AUC = 0.69 in GSE20271) and MammaPrint (lowest AUC = 0.69 in GSE20271) (Table S6). However, the predictive ability of RPS in ER‐negative patients was relatively lower compared to its prediction ability in ER‐positive patients with the lowest AUC = 0.64 in the Hess *et al* dataset (Figure 2F and Table S6). In summary, we validated the efficacy of our computational framework by showing the RPS’s predictive power in breast cancer, particularly its comparative prediction power with commercialized signatures in ER‐positive patients.

### 3.3 The TNBC‐RPS predicts NCT response in TNBC patients

**FIGURE 3:**
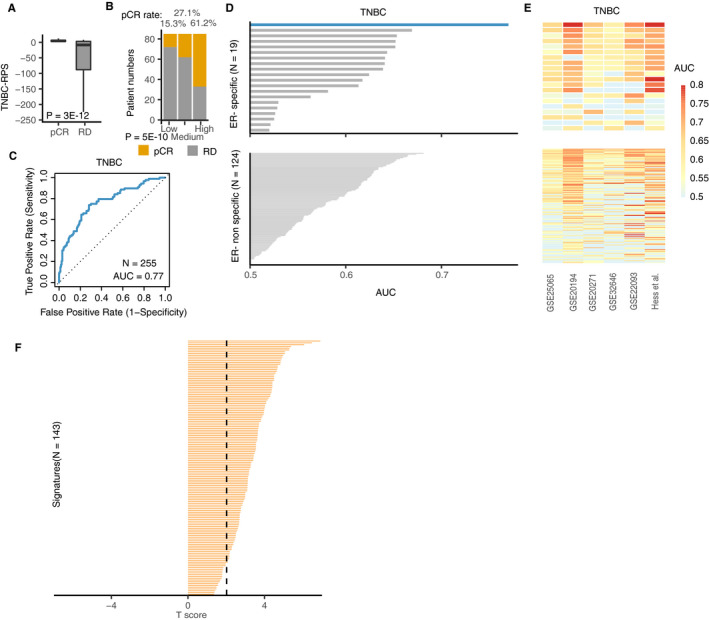
The TNBC‐RPS predicts NCT response in TNBC patients. (A) The TNBC‐RPS is higher in pCR than RD samples. The statistical significance is calculated by Wilcoxon rank sum test; (**B)** pCR patients are enriched in the high‐TNBC‐RPS group. TNBC patients are separated based on the tertile of their TNBC‐RPS. The pCR patients are significantly enriched in the high‐TNBC‐RPS group. The statistical significance is calculated by Chi‐square test; (**C)** The ROC curve for pCR prediction in TNBC patients using the TNBC‐RPS as a feature; (**D)** Comparison of the TNBC‐RPS with the prediction power of other public signatures in TNBC patients. 143 signatures are separated into ER‐negative‐ and non‐ER‐negative‐specific predictive signature. Barplot shows the area under the curve (AUC) difference between the TNBC‐RPS and other public signatures in TNBC patients; (**E)** Comparison of the TNBC‐RPS with the prediction power of other public signatures prediction power in TNBC patients across six individual datasets. “Up” panel is corresponding to ER‐negative‐specific signatures and “down” panel is corresponding to non‐ER‐specific signatures; (**F)** *T* statistics show the AUC difference between the TNBC‐RPS and other signatures in predicting response. Dashed line indicates the statistical cut‐off (*P* < .05)

After showing the efficacy of the framework in ER‐positive breast cancer, we aimed to define a signature that could predict NCT response of ER‐negative patients. Here, we focused on triple‐negative breast cancer (TNBC), an aggressive and heterogeneous subtype. No clinically practical signatures are currently available for predicting patient response to NCT in these patients. We applied our framework to TNBC patients in the previous training dataset (GSE25055) and built a TNBC‐specific signature to capture gene expression differences between pCR and RD of TNBC patients. Unsurprisingly, the TNBC‐RPS calculated in the training dataset is predictive of NCT response (*P* = 5e‐11, AUC = 0.87, Figure S3C‐D). We then integrated the TNBC‐RPS signature with TNBC patient expression profile in the validation metadata to calculate the TNBC‐RPSs. The pCR patients had significantly higher TNBC‐RPS than RD patients (*P* = 3e‐12, Figure 3A). Moreover, the pCR patients were significantly enriched in the high‐TNBC‐RPS group compared to other groups, with a pCR rate of 61.2% compared to a baseline pCR rate of 33.2% (*P* = 5e‐10, Figure 3B). We further quantified the predictive power of the TNBC‐RPS in TNBC patients from our metadata set and observed an AUC = 0.77 (Figure 3C). Also, the prediction power of TNBC‐RPS could be observed in each individual dataset (Table S7 and S8).

The performance of the TNBC‐RPS to predict NCT response was compared to previously defined predictive signatures. As stated in the previous section, we collected 143 predictive signatures, which were composed of ER‐positive‐specific signatures, ER‐negative‐specific signatures, and nonspecific signatures. From these, 19 ER‐negative‐specific signatures were identified, and the other 124 signatures were grouped into ER‐negative‐non‐specific signatures for comparison. As shown in the upper panel of Figure 3D, the TNBC‐RPS outperformed 19 ER‐negative‐specific predictive signatures in predicting NCT response, with an AUC of 0.77. The next‐highest AUC was achieved by the loss of *PTEN* gene signature (AUC = 0.67, Table S7), which has been reported to predict NCT response in TNBC patients.^28^, ^58^ The TNBC‐RPS also outperformed all ER‐negative‐nonspecific predictive signatures in the validation metadata (Figure 3D and Table S7). The highest AUC of the ER‐negative‐nonspecific predictive signature was achieved by an *E2F1* pathway‐related gene signature (AUC = 0.68, Table S7). We further examined the AUC of each signature across the individual dataset (Figure 3E) and found that the TNBC‐RPS was predictive of NCT response in TNBC patients across all datasets, with the highest AUC = 0.91 in GSE20194 and Hess *et al* dataset (Table S7). In three of six datasets, the TNBC‐RPS had an AUC higher than 0.75 while other signatures did not present such consistent prediction power (Figure 3E and Table S7). To compare the predictive accuracy between the TNBC‐RPS and other signatures, a paired T‐test was used to measure the statistical significance of AUC differences across six individual datasets. As shown in Figure 3F, the TNBC‐RPS significantly outperformed 129 of 143 signatures in predicting NCT response (*P* < .05, Figure 3F). In cases in which the TNBC‐RPS was not statistically significant compared to other signatures, a positive trend in the T‐score was observed; this still indicates a better prediction ability when using the TNBC‐RPS as the predictor.

### 3.4 The TNBC‐RPS predicts NCT response in each clinical stage and grade

**FIGURE 4:**
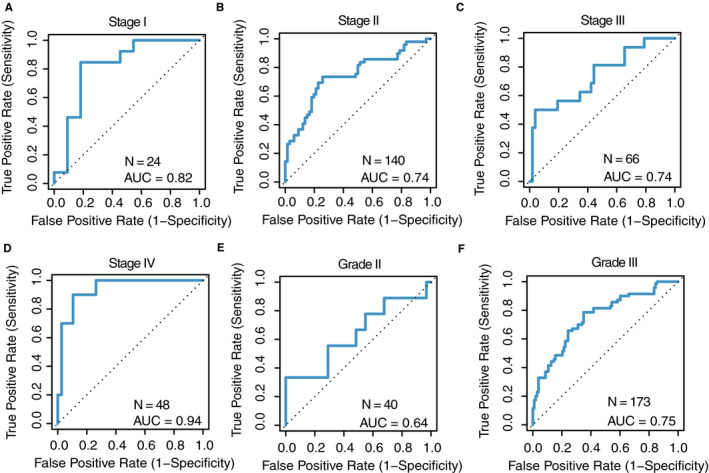
The TNBC‐RPS predicts NCT response in each stage and grade. (A) Stage I; (B) Stage II; (C) Stage III; (D) Stage IV; (E) Grade II; and (F) Grade III. Receiver Operating Characteristic (ROC) curves for pCR prediction using the TNBC‐RPS as feature

Although the TNBC‐RPS showed good prediction power in TNBC patients, we were concerned that tumor stage or grade might have confounded these findings; it has been reported that TNBC patients with a more advanced tumor stage or grade tend to have better response to NCT.^59^ To evaluate this, we first examined the predictive ability of the TNBC‐RPS in the validation metadata for each tumor stage. By calculating the TNBC‐RPS for each individual stage in both pCR and RD patients (Table S9), we found that pCR patients had significantly higher RPS than RD patients (*P* = .007, Stage I; *P* = 9e‐6, Stage II; *P* = .004, Stage III; *P* = 1e‐6, Stage IV; Table S9). Secondly, we calculated the AUC of the TNBC‐RPS in a stage‐specific manner. As shown in Figure 4, the TNBC‐RPS could predict stage‐specific responses in TNBC patients, indicating that the predictive power was not affected by stage stratification (Figure 4A‐D). Interestingly, the TNBC‐RPS showed a high predictive power within stage‐I patients (AUC = 0.82, TNBC‐RPS; Figure 4A), indicating that the TNBC‐RPS could robustly predict NCT response in early‐stage breast cancer patients. This is important since the development of diagnostic techniques increases the number of patients diagnosed at early stages, thus requiring predictive markers that are effect at those stages.

Similar to tumor stage, tumor grade may also confound the prediction of the TNBC‐RPS in TNBC patients. Hence, we performed the same analyses by using the TNBC‐RPS as the predictor in each tumor grade and found that AUC = 0.64 and AUC = 0.75 in grade‐II and grade‐III patients (Figure 4E‐F) respectively. The conclusion of the TNBC‐RPS being a grade‐specific predictor was further validated by comparing the TNBC‐RPS difference between pCR and RD patients (Table S9).

### 3.5 The TNBC‐PRS adds additional predictive power to current clinical predictors

**FIGURE 5:**
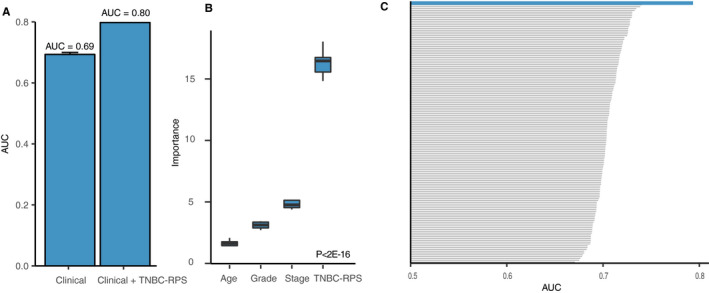
The TNBC‐RPS provides additional information to current clinical predictors in prediction. (A) The TNBC‐RPS provides additional information over current clinical predictors in TNBC patients. Barplot shows the difference of the AUC by using clinical predictors and using the combination of clinical predictors and the TNBC‐RPS. Error bars indicate the standard deviation calculated by performing 10‐fold cross‐validation 100 times; (**B)** The TNBC‐RPS is dominant in the prediction process in TNBC patients. Boxplot shows the relative importance difference of the TNBC‐RPS and other clinical predictors in the pCR classification model. *P*‐value was calculated by analysis of variance (ANOVA); (**C)** The comparison of the TNBC‐RPS with the predictive power of 143 signatures in TNBC patients. Barplot shows the area under the curve (AUC) difference between the TNBC‐RPS and other signatures in TNBC patients in the pCR classification model combined with clinical predictors

We have demonstrated that the TNBC‐RPS could predict pCR in each TNBC clinical stage and grade. In clinical practice, the combination of clinical stage, grade, and age is used to predict NCT response.^60^ Therefore, we investigated whether adding the TNBC‐RPS to current clinical predictors could further improve prediction accuracy. First, we applied Random Forest algorithm to calculate AUC for clinical predictors in TNBC patients and performed 10‐fold cross‐validation. As shown in Figure 5A, the prediction accuracy only achieved an AUC of 0.69 with the use of clinical predictors. Then, by adding the TNBC‐RPS to clinical predictors, we improved the AUC to 0.80 (Table S10). In order to further understand the contribution of the TNBC‐RPS to the prediction, we investigated the relative importance of the TNBC‐RPS and clinical factors through a 10‐fold cross validation process. As expected, the relative importance of the TNBC‐RPS was significantly higher than other clinical predictors, which indicated the predominant predictive power of the TNBC‐RPS in the TNBC NCT response prediction (*P* < 2e‐16, Figure 5B).

Next, we assessed whether other gene expression signatures displayed similar properties. Thus, we repeated the same analysis as mentioned above and combined clinical predictors with each of the 143 signatures to test the AUC change. As shown in Figure 5C, 90 of 143 signatures had an AUC higher than 0.7, with TNBC‐RPS having the highest AUC = 0.80. The second‐highest signature was reported by Witklewicz *et al*, with an AUC = 0.74 (Table S10). In summary, the TNBC‐RPS combined with the clinical predictors outperformed the prediction accuracy compared to the previous signatures (AUC = 0.80, Figure 5C and Table S10). In addition, we also performed the same analyses by using RPS in ER‐positive patients and found that RPS could further improve the prediction accuracy of the current clinical predictors to AUC = 0.79 (Table S11).

### 3.6 The TNBC‐RPS associates with immune cell infiltration, stromal cell abundance, and cell proliferation

**FIGURE 6:**
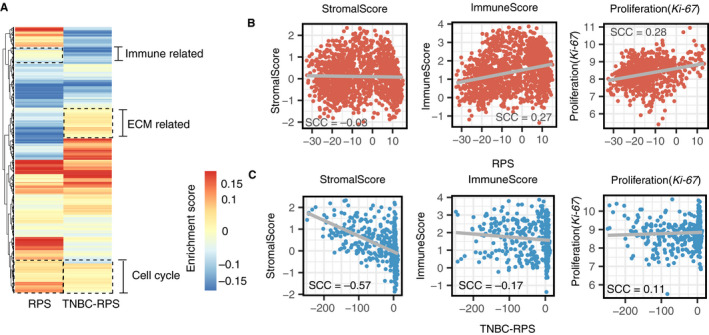
The RPS and TNBC‐RPS capture the tumor microenvironment characteristics. (A) Pathway enrichment test of the RPS and TNBC‐RPS gene signatures; (**B)** The RPS correlates with immune cell abundance and tumor proliferation rate in ER‐positive patients; (**C)** The TNBC‐RPS correlates with stromal cell abundance, immune cell infiltration, and tumor proliferation rate in TNBC patients. The Spearman correlation coefficient (SCC) is calculated by Spearman correlation

To biologically annotated RPS and TNBC‐RPS, which could potentially indicate the different mechanisms underlying the chemotherapeutic response between ER‐positive and TNBC patients, we performed a pathway enrichment analysis in both gene signatures (Figure 6A and Table S12). A positive enrichment score refers to the enrichment of pathways in the up‐regulated genes of the signature, while a negative enrichment score refers to the enrichment of pathways in the down‐regulated genes of signature. Interestingly, we found that some pathways were shared in both signatures, while some pathways were presented in a signature‐specific way. Cell‐cycle‐related pathways were shared between the RPS and TNBC‐RPS, indicating the involvement of cell‐cycle pathways in the NCT response. For example, the KEGG cell cycle pathway was shared by the gene signatures of the RPS and TNBC‐PRS, with an enrichment score 0.20 and 0.16 respectively (Table S12). Moreover, pathways related to immune response were also found in both the RPS and TNBC‐RPS. The REACTOME antigen‐presenting pathways were enriched in the TNBC‐RPS gene signature (with enrichment scores of −0.12) and the KEGG T‐cell‐receptor‐related pathways were enriched in the RPS gene signature (with enrichment scores of −0.11). In addition to shared pathways, we also identified signature‐specific pathways. For example, protein transportation‐related pathways, like the REACTOME Extracellular Matrix (ECM) pathway, were only enriched in the TNBC‐RPS gene signature (with an enrichment score of 0.11) (Table S12).

We next performed clustering analysis on the pathway‐enrichment scores in the RPS and TNBC‐RPS to better compare these two signatures (Figure 6A). The enriched cell‐cycle and immune‐related pathways in both the RPS and TNBC‐RPS gene signatures clustered together, while the enriched ECM pathways only formed a unique cluster in the TNBC‐RPS gene signature. Moreover, by performing gene ontology (GO) enrichment analysis in the RPS and TNBC‐RPS gene signatures, we validated biological processes that had been identified by the pathway analyses (Tables S13 and S14). From both the pathway and GO enrichment analyses, we hypothesized that the RPS and TNBC‐RPS could capture tumor microenvironment characteristics, in which the RPS reflects both the cell‐cycle and immune‐related pathway activities, while the TNBC‐RPS reflects the activities of pathways including the cell‐cycle, immune‐related, and ECM pathways.

To examine our hypothesis, we deconvoluted the tumor microenvironment into three general components—infiltrating immune cell abundance, stromal cell abundance, and tumor cell proliferation rate—to represent the activation of immune‐related, ECM‐related, and cell‐proliferation‐related pathways (see methods) respectively. The association between the RPS and those three components in ER‐positive patients was examined by Spearman correlation. The RPS was positively correlated with immune cell infiltration and tumor cell proliferation rate (SCC = 0.27, immune cell infiltration; SCC = 0.28, tumor cell proliferation rate, Figure 6B), but was not associated with stromal cell abundance (SCC = 0.03, Figure 6B). Moreover, we performed similar analyses using the TNBC‐RPS and demonstrated that it was strongly negatively correlated with stromal cell abundance and was weakly correlated with immune cell infiltration and tumor cell proliferation rate (SCC = −0.57, stromal cell abundance; SCC = −0.17, immune cell infiltration; SCC = 0.11, tumor cell proliferation rate; Figure 6C). Given the fact that the TNBC‐RPS mainly correlated with stromal cell abundance, we examined if stromal cell abundance could be used for prediction. We found that stromal cell abundance was predictive for the NCT in TNBC patients, with an AUC = 0.55 (Figure S3E‐F).

## 4 DISCUSSION

Neoadjuvant chemotherapy is being used more and more frequently for treating breast cancer patients. This is due to its advantages in reducing tumor size, improving surgical options, and significantly increasing survival in responders. However, broad clinical application remains questionable because of a low response rate and the potential for significant side effects. The most extreme case is TNBC, which is the most aggressive subtype of breast cancer and has the worst prognostic outcome. Due to its heterogeneity, patients with TNBC respond differently to NCT. Numerous efforts have been put into developing the predictive signatures for TNBC, but, currently, there is no clinically applied predictive signature. Therefore, there is an urgent need for developing robust predictive biomarkers for TNBC patients. Although many studies have focused on the chemotherapy regulatory program difference between pCR and RD,^61^, ^62^, ^63^ the mechanisms underlying the survival of resistant tumor cells remain poorly understood.

In this study, we developed a novel framework for identifying predictive gene signatures in breast cancer patients. We validated the efficacy of this framework by showing that the RPS predicted NCT response in breast cancer patients, particularly in ER‐positive patients (Figure 2A‐C and Table S6). In addition, compared to the commercialized signatures, the RPS had a comparable prediction ability across each individual dataset (Figure 2D‐E and Table S6). We then applied the framework to TNBC patients and calculated the TNBC‐RPS. The TNBC‐RPS was predictive of the response in TNBC patients (Figure 3A‐C and Table S7). Compared to the previously‐developed ER‐negative‐specific and nonspecific prediction signatures, the TNBC‐RPS outperformed 143 predictive gene signatures and presented robust prediction accuracy (Figure 3D‐F and Table S7). Of importance, the TNBC‐RPS leads to a higher AUC of up to 0.80 in TNBC patients (Figure 5A‐B) and exceeded the performance of the 143 predictive gene signatures when combined with clinical predictors (Figure 5C). We, therefore, provide a new framework for identifying predictive markers of NCT response. In addition, to facilitate the clinical utility of RPS and TNBC‐RPS signatures, we also provided a revised version of those two gene signatures with fewer genes (Table S15).

Previous studies calculated the scores of different gene signatures using only a single method. This strategy does not take into account the variation in the methods used to calculate the scores from the gene signatures. Instead of using this one‐method‐fits‐all strategy, we validated the previously published signatures by applying the same algorithms that were used to calculate the scores of each signature to the same datasets and reproduced the published prediction performances. Then, we applied the gene signatures to the validation metadata for prediction. This made the prediction accuracy comparison more objective since we took the impact of different methods into consideration (Figures 2 and 3). In Table S4, we present the validation results of the previous signatures. We acquired consistent results by repeating previous published gene signatures in their validation datasets. Despite the subtle differences between the *P*‐value reported previously and our calculated AUC (likely caused by the update or different normalization methods on the raw microarray data), we showed that our model significantly outperformed most of the reported signatures.

The drug‐response mechanisms in breast cancer have been studied for many years but were still poorly understood. We investigated the association between the RPS and characteristics of the ER‐positive tumor microenvironment, as well as between the TNBC‐RPS and characteristics of the TNBC tumor microenvironment respectively (Figure 6). Of note, the RPS identified changes in the tumor cell proliferation rate and immune cell infiltration in ER‐positive patients, which was supported by previous studies showing that the cell‐cycle‐related^16^, ^64^, ^65^, ^66^ and immune‐infiltration‐related gene signatures^67^, ^68^, ^69^ were associated with responsiveness. This observation could be further validated through the prediction performance of the 143 predictive gene signatures. For example, Oncotype DX, a signature composed of cell‐cycle‐related genes, and the Immune Signature Gene Module score were both predictive to the response in ER‐positive patients (Table S6).^16^, ^28^ The TNBC‐RPS primarily captured the relative abundance of the stromal cells in the tumor microenvironment. Farmer *et al* reported the similar finding in TNBC patients, as well.^29^ Meanwhile, we also used the stromal cell abundance for prediction in TNBC patients and got an AUC = 0.55, indicating a predictive role of stromal cells in TNBC patients’ NCT response (Figure S3E‐F).^69^, ^70^, ^71^, ^72^ Therefore, our findings provide an understanding of cancer biology in breast cancer by showing which aspect(s) of the tumor microenvironment might influence the response to the NCT.

Although we have demonstrated the efficacy of the RPS and the TNBC‐RPS in predicting the response to NCT, the prediction power and the applicable range of the model could be further improved. In addition to the gene signatures, other IHC‐staining signatures or MRI imaging‐based prediction models were used to predict the response to NCT.^73^, ^74^, ^75^ However, we lack the data to compare the performance of our signatures to these methods or to integrate them into the model for better prediction. Moreover, our signatures were applicable to the prediction of the combination of antimetabolite‐, anthracycline‐, alkylating agent‐, and taxane‐based chemotherapy‐treated patients and have not been extended to investigate its predictive power with other chemotherapy agents or targeted therapy agents. With the release of more gene expression data, it may be possible to extend the applicable range of our signatures or to develop drug‐specific‐predictive gene signatures.

In summary, we developed a framework for identifying a predictive gene signature in breast cancer and defined two gene signatures that could be used to predict NCT response in ER‐positive and TNBC patients respectively. We have demonstrated that the RPS performed at a comparable level to the current commercialized signatures, while the TNBC‐RPS outperformed 143 gene signatures for TNBC patients in prediction. More importantly, integrating the RPS or TNBC‐RPS with current established clinical predictors enhanced the predictive power, compared to using the clinical predictors only. In addition, the RPS and TNBC‐RPS captured different aspects of the tumor microenvironment, leading to tantalizing insights as to the potential biological mechanisms driving differences in the chemotherapeutic response. This computational framework can also be readily extended to define predictive biomarkers in other cancer types.

## CONFLICTS OF INTERESTS

The authors declare that they have no competing interests.

## AUTHOR CONTRIBUTION

Conception and design: YZ and CC. Development of methodology: YZ and CC. Acquisition of data: YZ and CC. Analysis and interpretation of data: YZ, ES, and CC. Writing, review, and/or revision of the manuscript: YZ, ES, and CC. All authors read approved the final manuscript.

## Supporting information

Supplementary Material

Supplementary Material

## Data Availability

The six Gene Expression Omnibus (https://www.ncbi.nlm.nih.gov/geo/) datasets analyzed in this study are under the following accession numbers: GSE25055, GSE20194, GSE25065, GSE20271, GSE32646, and GSE22093. Hess *et al* dataset gene expression dataset is downloaded from MD Anderson Cancer Center public database (https://bioinformatics.mdanderson.org/public‐datasets/).

## References

[1] Liu SV , Melstrom L , Yao K , Russell CA , Sener SF . Neoadjuvant therapy for breast cancer. J Surg Oncol. 2010;101(4):283‐291. 20187061 10.1002/jso.21446

[2] Fisher B , Bryant J , Wolmark N , et al. Effect of preoperative chemotherapy on the outcome of women with operable breast cancer. J Clin Oncol Off J Am Soc Clin Oncol. 1998;16(8):2672‐2685. 10.1200/JCO.1998.16.8.2672 9704717

[3] Rastogi P , Anderson SJ , Bear HD , et al. Preoperative chemotherapy: updates of National Surgical Adjuvant Breast and Bowel Project Protocols B‐18 and B‐27. J Clin Oncol Off J Am Soc Clin Oncol. 2008;26(5):778‐785. 10.1200/JCO.2007.15.0235 18258986

[4] Mauri D , Pavlidis N , Ioannidis JPA . Neoadjuvant versus adjuvant systemic treatment in breast cancer: a meta‐analysis. J Natl Cancer Inst. 2005;97(3):188‐194. 15687361 10.1093/jnci/dji021

[5] Kaufmann M , von Minckwitz G , Mamounas EP , et al. Recommendations from an international consensus conference on the current status and future of neoadjuvant systemic therapy in primary breast cancer. Ann Surg Oncol. 2012;19(5):1508‐1516. 22193884 10.1245/s10434-011-2108-2

[6] Vaidya JS , Massarut S , Vaidya HJ , et al. Rethinking neoadjuvant chemotherapy for breast cancer. BMJ. 2018;11(360):j5913. 10.1136/bmj.j5913 29326104

[7] Bonadonna G , Valagussa P , Brambilla C , et al. Primary chemotherapy in operable breast cancer: eight‐year experience at the Milan Cancer Institute. J Clin Oncol Off J Am Soc Clin Oncol. 1998;16(1):93‐100. 10.1200/JCO.1998.16.1.93 9440728

[8] Paik S , Shak S , Tang G , et al. A multigene assay to predict recurrence of tamoxifen‐treated, node‐negative breast cancer. N Engl J Med. 2004;351(27):2817‐2826. 15591335 10.1056/NEJMoa041588

[9] van 't Veer LJ , Dai H , van de Vijver MJ , et al. Gene expression profiling predicts clinical outcome of breast cancer. Nature 2002;415(6871):530–536. 11823860 10.1038/415530a

[10] van de Vijver MJ , He YD , van 't Veer LJ , et al. A gene‐expression signature as a predictor of survival in breast cancer. N Engl J Med. 2002;347(25):1999–2009. 12490681 10.1056/NEJMoa021967

[11] Chang JC , Makris A , Gutierrez MC , et al. Gene expression patterns in formalin‐fixed, paraffin‐embedded core biopsies predict docetaxel chemosensitivity in breast cancer patients. Breast Cancer Res Treat. 2008;108(2):233‐240. 17468949 10.1007/s10549-007-9590-z

[12] Knauer M , Mook S , Rutgers EJT , et al. The predictive value of the 70‐gene signature for adjuvant chemotherapy in early breast cancer. Breast Cancer Res Treat. 2010;120(3):655‐661. 20204499 10.1007/s10549-010-0814-2

[13] Ross JS , Hatzis C , Symmans WF , Pusztai L , Hortobágyi GN . Commercialized multigene predictors of clinical outcome for breast cancer. Oncologist. 2008;13(5):477‐493. 18515733 10.1634/theoncologist.2007-0248

[14] Nicolini A , Ferrari P , Duffy MJ . Prognostic and predictive biomarkers in breast cancer: past, present and future. Semin Cancer Biol. 2018;52(Pt 1):56‐73. 28882552 10.1016/j.semcancer.2017.08.010

[15] Markopoulos C , van de Velde C , Zarca D , Ozmen V , Masetti R . Clinical evidence supporting genomic tests in early breast cancer: do all genomic tests provide the same information? Eur J Surg Oncol J Eur Soc Surg Oncol Br Assoc Surg Oncol. 2017;43(5):909‐920. 10.1016/j.ejso.2016.08.012 27639633

[16] Bear HD , Wan W , Robidoux A , et al. Using the 21‐gene assay from core needle biopsies to choose neoadjuvant therapy for breast cancer: a multicenter trial. J Surg Oncol. 2017;115(8):917‐923. 28407247 10.1002/jso.24610 PMC5481477

[17] Gianni L , Zambetti M , Clark K , et al. Gene expression profiles in paraffin‐embedded core biopsy tissue predict response to chemotherapy in women with locally advanced breast cancer. J Clin Oncol Off J Am Soc Clin Oncol. 2005;23(29):7265‐7277. 10.1200/JCO.2005.02.0818 16145055

[18] Yardley DA , Peacock NW , Shastry M , et al. A phase II trial of ixabepilone and cyclophosphamide as neoadjuvant therapy for patients with HER2‐negative breast cancer: correlation of pathologic complete response with the 21‐gene recurrence score. Breast Cancer Res Treat. 2015;154(2):299‐308. 26507191 10.1007/s10549-015-3613-y

[19] Bertucci F , Finetti P , Viens P , Birnbaum D . EndoPredict predicts for the response to neoadjuvant chemotherapy in ER‐positive, HER2‐negative breast cancer. Cancer Lett. 2014;355(1):70‐75. 25218596 10.1016/j.canlet.2014.09.014

[20] Prat A , Galvan P , Jimenez B , et al. Prediction of response to Neoadjuvant chemotherapy using core needle biopsy samples with the Prosigna Assay. Clin Cancer Res Off J Am Assoc Cancer Res. 2016;22(3):560‐566. 10.1158/1078-0432.CCR-15-0630 26152740

[21] Dent R , Trudeau M , Pritchard KI , et al. Triple‐negative breast cancer: clinical features and patterns of recurrence. Clin Cancer Res Off J Am Assoc Cancer Res. 2007;13(15 Pt 1):4429‐4434. 10.1158/1078-0432.CCR-06-3045 17671126

[22] Venkitaraman R . Triple‐negative/basal‐like breast cancer: clinical, pathologic and molecular features. Expert Rev Anticancer Ther. 2010;10(2):199‐207. 20131996 10.1586/era.09.189

[23] Carey L , Winer E , Viale G , Cameron D , Gianni L . Triple‐negative breast cancer: disease entity or title of convenience? Nat Rev Clin Oncol. 2010;7(12):683‐692. 20877296 10.1038/nrclinonc.2010.154

[24] Yin W‐J , Lu J‐S , Di G‐H , et al. Clinicopathological features of the triple‐negative tumors in Chinese breast cancer patients. Breast Cancer Res Treat. 2009;115(2):325‐333. 18563552 10.1007/s10549-008-0096-0

[25] Lee J‐M , Ledermann JA , Kohn EC . PARP Inhibitors for BRCA1/2 mutation‐associated and BRCA‐like malignancies. Ann Oncol Off J Eur Soc Med Oncol. 2014;25(1):32‐40. 10.1093/annonc/mdt384 PMC3868320 24225019

[26] McAndrew N , DeMichele A . Neoadjuvant chemotherapy considerations in triple‐negative breast cancer. J Target Ther Cancer. 2018;7(1):52‐69. 29577076 PMC5865448

[27] Gingras I , Desmedt C , Ignatiadis M , Sotiriou C . CCR 20th anniversary commentary: gene‐expression signature in breast cancer‐where did it start and where are we now? Clin Cancer Res Off J Am Assoc Cancer Res. 2015;21(21):4743‐4746. 10.1158/1078-0432.CCR-14-3127 26527804

[28] Ignatiadis M , Singhal SK , Desmedt C , et al. Gene modules and response to neoadjuvant chemotherapy in breast cancer subtypes: a pooled analysis. J Clin Oncol Off J Am Soc Clin Oncol. 2012;30(16):1996‐2004. 10.1200/JCO.2011.39.5624 22508827

[29] Farmer P , Bonnefoi H , Anderle P , et al. A stroma‐related gene signature predicts resistance to neoadjuvant chemotherapy in breast cancer. Nat Med. 2009;15(1):68‐74. 19122658 10.1038/nm.1908

[30] Chen Y‐Z , Kim Y , Soliman HH , Ying G , Lee JK . Single drug biomarker prediction for ER‐ breast cancer outcome from chemotherapy. Endocr Relat Cancer. 2018;25(6):595‐605. 29599124 10.1530/ERC-17-0495 PMC5920016

[31] Hatzis C , Pusztai L , Valero V , et al. A genomic predictor of response and survival following taxane‐anthracycline chemotherapy for invasive breast cancer. JAMA. 2011;305(18):1873‐1881. 21558518 10.1001/jama.2011.593 PMC5638042

[32] Shi L , Campbell G , Jones WD , et al. The MicroArray Quality Control (MAQC)‐II study of common practices for the development and validation of microarray‐based predictive models. Nat Biotechnol. 2010;28(8):827‐838. 20676074 10.1038/nbt.1665 PMC3315840

[33] Tabchy A , Valero V , Vidaurre T , et al. Evaluation of a 30‐gene paclitaxel, fluorouracil, doxorubicin, and cyclophosphamide chemotherapy response predictor in a multicenter randomized trial in breast cancer. Clin Cancer Res Off J Am Assoc Cancer Res. 2010;16(21):5351‐5361. 10.1158/1078-0432.CCR-10-1265 PMC4181852 20829329

[34] Hess KR , Anderson K , Symmans WF , et al. Pharmacogenomic predictor of sensitivity to preoperative chemotherapy with paclitaxel and fluorouracil, doxorubicin, and cyclophosphamide in breast cancer. J Clin Oncol Off J Am Soc Clin Oncol. 2006;24(26):4236‐4244. 10.1200/JCO.2006.05.6861 16896004

[35] Miyake T , Nakayama T , Naoi Y , et al. GSTP1 expression predicts poor pathological complete response to neoadjuvant chemotherapy in ER‐negative breast cancer. Cancer Sci. 2012;103(5):913‐920. 22320227 10.1111/j.1349-7006.2012.02231.x PMC7659189

[36] Iwamoto T , Bianchini G , Booser D , et al. pathways associated with prognosis and chemotherapy sensitivity in molecular subtypes of breast cancer. J Natl Cancer Inst. 2011;103(3):264‐272. 21191116 10.1093/jnci/djq524

[37] Leek JT , Johnson WE , Parker HS , Jaffe AE , Storey JD . The sva package for removing batch effects and other unwanted variation in high‐throughput experiments. Bioinforma Oxf Engl. 2012;28(6):882‐883. 10.1093/bioinformatics/bts034 PMC3307112 22257669

[38] Cheng C , Yan X , Sun F , Li LM . Inferring activity changes of transcription factors by binding association with sorted expression profiles. BMC Bioinformati. 2007;16(8):452. 10.1186/1471-2105-8-452 PMC2194743 18021409

[39] Zhao Y , Varn FS , Cai G , Xiao F , Amos CI , Cheng C . A P53‐deficiency gene signature predicts recurrence risk of patients with early‐stage lung adenocarcinoma. Cancer Epidemiol Biomark Prev Publ Am Assoc Cancer Res Cosponsored Am Soc Prev Oncol. 2018;27(1):86‐95. 10.1158/1055-9965.EPI-17-0478 PMC5839302 29141854

[40] Schaafsma E , Zhao Y , Wang Y , et al. Whole transcriptome signature for prognostic prediction (WTSPP): application of whole transcriptome signature for prognostic prediction in cancer. Lab Investig J Tech Methods Pathol. 2020;6. 10.1038/s41374-020-0413-8 PMC7483260 32144347

[41] Zhao Y , Carter R , Natarajan S , et al. Single‐cell RNA sequencing reveals the impact of chromosomal instability on glioblastoma cancer stem cells. BMC Med Genomics. 2019;12(1):79. 31151460 10.1186/s12920-019-0532-5 PMC6545015

[42] Schaafsma E , Yuan Y , Zhao Y , Cheng C . Computational STAT3 activity inference reveals its roles in the pancreatic tumor microenvironment. Sci Rep. 2019;9(1):18257. 31796877 10.1038/s41598-019-54791-x PMC6890662

[43] Filipits M , Rudas M , Jakesz R , et al. A new molecular predictor of distant recurrence in ER‐positive, HER2‐negative breast cancer adds independent information to conventional clinical risk factors. Clin Cancer Res Off J Am Assoc Cancer Res. 2011;17(18):6012‐6020. 10.1158/1078-0432.CCR-11-0926 21807638

[44] Wang Y , Klijn JGM , Zhang YI , et al. Gene‐expression profiles to predict distant metastasis of lymph‐node‐negative primary breast cancer. Lancet Lond Engl. 2005;365(9460):671‐679. 10.1016/S0140-6736(05)17947-1 15721472

[45] Sotiriou C , Wirapati P , Loi S , et al. Gene expression profiling in breast cancer: understanding the molecular basis of histologic grade to improve prognosis. J Natl Cancer Inst. 2006;98(4):262‐272. 16478745 10.1093/jnci/djj052

[46] Parker JS , Mullins M , Cheang MCU , et al. Supervised risk predictor of breast cancer based on intrinsic subtypes. J Clin Oncol Off J Am Soc Clin Oncol. 2009;27(8):1160‐1167. 10.1200/JCO.2008.18.1370 PMC2667820 19204204

[47] Gendoo DMA , Ratanasirigulchai N , Schröder MS , et al. Genefu: an R/Bioconductor package for computation of gene expression‐based signatures in breast cancer. Bioinforma Oxf Engl. 2016;32(7):1097‐1099. 10.1093/bioinformatics/btv693 PMC6410906 26607490

[48] Stover DG , Coloff JL , Barry WT , Brugge JS , Winer EP , Selfors LM . The role of proliferation in determining response to neoadjuvant chemotherapy in breast cancer: a gene expression‐based meta‐analysis. Clin Cancer Res Off J Am Assoc Cancer Res. 2016;22(24):6039‐6050. 10.1158/1078-0432.CCR-16-0471 PMC5161615 27330058

[49] Witkiewicz AK , Balaji U , Knudsen ES . Systematically defining single‐gene determinants of response to neoadjuvant chemotherapy reveals specific biomarkers. Clin Cancer Res Off J Am Assoc Cancer Res. 2014;20(18):4837‐4848. 10.1158/1078-0432.CCR-14-0885 PMC5286972 25047707

[50] Turner N , Forcato M , Nuzzo S , Malorni L , Bicciato S , Di Leo A . A multifactorial “Consensus Signature” by in silico analysis to predict response to neoadjuvant anthracycline‐based chemotherapy in triple‐negative breast cancer. NPJ Breast Cancer. 2015;1:15003. 28721363 10.1038/npjbcancer.2015.3 PMC5515202

[51] Desmedt C , Di Leo A , de Azambuja E , et al. Multifactorial approach to predicting resistance to anthracyclines. J Clin Oncol Off J Am Soc Clin Oncol. 2011;29(12):1578‐1586. 10.1200/JCO.2010.31.2231 21422418

[52] Juul N , Szallasi Z , Eklund AC , et al. Assessment of an RNA interference screen‐derived mitotic and ceramide pathway metagene as a predictor of response to neoadjuvant paclitaxel for primary triple‐negative breast cancer: a retrospective analysis of five clinical trials. Lancet Oncol. 2010;11(4):358‐365. 20189874 10.1016/S1470-2045(10)70018-8

[53] Breiman L . Random Forests. Mach Learn. 2001;45(1):5‐32.

[54] Subramanian A , Tamayo P , Mootha VK , et al. Gene set enrichment analysis: a knowledge‐based approach for interpreting genome‐wide expression profiles. Proc Natl Acad Sci U S A. 2005;102(43):15545‐15550. 16199517 10.1073/pnas.0506580102 PMC1239896

[55] Yoshihara K , Shahmoradgoli M , Martínez E , et al. Inferring tumour purity and stromal and immune cell admixture from expression data. Nat Commun. 2013;4:2612. 24113773 10.1038/ncomms3612 PMC3826632

[56] Gerdes J . Ki‐67 and other proliferation markers useful for immunohistological diagnostic and prognostic evaluations in human malignancies. Semin Cancer Biol. 1990;1(3):199‐206. 2103495

[57] Mougalian SS , Soulos PR , Killelea BK , et al. Use of neoadjuvant chemotherapy for patients with stage I to III breast cancer in the United States. Cancer. 2015;121(15):2544‐2552. 25902916 10.1002/cncr.29348

[58] Straver ME , Glas AM , Hannemann J , et al. The 70‐gene signature as a response predictor for neoadjuvant chemotherapy in breast cancer. Breast Cancer Res Treat. 2010;119(3):551‐558. 19214742 10.1007/s10549-009-0333-1

[59] Goorts B , van Nijnatten TJA , de Munck L , et al. Clinical tumor stage is the most important predictor of pathological complete response rate after neoadjuvant chemotherapy in breast cancer patients. Breast Cancer Res Treat. 2017;163(1):83‐91. 28205044 10.1007/s10549-017-4155-2 PMC5387027

[60] Early Breast Cancer . Trialists’ Collaborative Group (EBCTCG). Long‐term outcomes for neoadjuvant versus adjuvant chemotherapy in early breast cancer: meta‐analysis of individual patient data from ten randomised trials. Lancet Oncol. 2018;19(1):27‐39. 29242041 10.1016/S1470-2045(17)30777-5 PMC5757427

[61] DeMichele A , Yee D , Esserman L . Mechanisms of resistance to neoadjuvant chemotherapy in breast cancer. N Engl J Med. 2017;377(23):2287‐2289. 29211674 10.1056/NEJMcibr1711545

[62] Cleator S , Parton M , Dowsett M . The biology of neoadjuvant chemotherapy for breast cancer. Endocr Relat Cancer. 2002;9(3):183‐195. 12237246 10.1677/erc.0.0090183

[63] Shaked Y . The pro‐tumorigenic host response to cancer therapies. Nat Rev Cancer. 2019;19(12):667‐685. 31645711 10.1038/s41568-019-0209-6

[64] Kim KI , Lee KH , Kim TR , Chun YS , Lee TH , Park HK . Ki‐67 as a predictor of response to neoadjuvant chemotherapy in breast cancer patients. J Breast Cancer. 2014;17(1):40‐46. 24744796 10.4048/jbc.2014.17.1.40 PMC3988341

[65] Mark KMK , Varn FS , Ung MH , Qian F , Cheng C . The E2F4 prognostic signature predicts pathological response to neoadjuvant chemotherapy in breast cancer patients. BMC Cancer. 2017;17(1):306. 28464832 10.1186/s12885-017-3297-2 PMC5414335

[66] Alba E , Lluch A , Ribelles N , et al. High proliferation predicts pathological complete response to neoadjuvant chemotherapy in early breast cancer. Oncologist. 2016;21(2):150‐155. 26786263 10.1634/theoncologist.2015-0312 PMC4746087

[67] García‐Martínez E , Gil GL , Benito AC , et al. Tumor‐infiltrating immune cell profiles and their change after neoadjuvant chemotherapy predict response and prognosis of breast cancer. Breast Cancer Res BCR. 2014;16(6):488. 25432519 10.1186/s13058-014-0488-5 PMC4303200

[68] Lee HJ , Seo J‐Y , Ahn J‐H , Ahn S‐H , Gong G . Tumor‐associated lymphocytes predict response to neoadjuvant chemotherapy in breast cancer patients. J Breast Cancer. 2013;16(1):32‐39. 23593079 10.4048/jbc.2013.16.1.32 PMC3625767

[69] Denkert C , Loibl S , Noske A , et al. Tumor‐associated lymphocytes as an independent predictor of response to neoadjuvant chemotherapy in breast cancer. J Clin Oncol Off J Am Soc Clin Oncol. 2010;28(1):105‐113. 10.1200/JCO.2009.23.7370 19917869

[70] Katayama MLH , Vieira RAdC , Andrade VP , et al. Stromal Cell Signature Associated with Response to Neoadjuvant Chemotherapy in Locally Advanced Breast Cancer. Cells. 2019;8(12). 10.3390/cells8121566 PMC6953077 31817155

[71] Nakasone E , Askautrud H , Kees T , et al. Imaging tumor‐stroma interactions during chemotherapy reveals contributions of the microenvironment to resistance. Cancer Cell. 2012;21(4):488‐503. 22516258 10.1016/j.ccr.2012.02.017 PMC3332002

[72] Wang Y , Brodsky AS , Xiong J , Lopresti ML , Yang D , Resnick MB . Stromal clusterin expression predicts therapeutic response to neoadjuvant chemotherapy in triple negative breast cancer. Clin Breast Cancer. 2018;18(3):e373‐e379. 28890185 10.1016/j.clbc.2017.08.007

[73] Price ER , Wong J , Mukhtar R , Hylton N , Esserman LJ . How to use magnetic resonance imaging following neoadjuvant chemotherapy in locally advanced breast cancer. World J Clin Cases. 2015;3(7):607‐613. 26244152 10.12998/wjcc.v3.i7.607 PMC4517335

[74] Goorts B , Dreuning KMA , Houwers JB , et al. MRI‐based response patterns during neoadjuvant chemotherapy can predict pathological (complete) response in patients with breast cancer. Breast Cancer Res BCR. 2018;20(1):34. 29669584 10.1186/s13058-018-0950-x PMC5907188

[75] Sjöström J . Predictive factors for response to chemotherapy in advanced breast cancer. Acta Oncol Stockh Swed. 2002;41(4):334‐345. 10.1080/028418602760169370 12234024

