# Supplementary material for: Gene signature‐based prediction of triple‐negative breast cancer patient response to Neoadjuvant chemotherapy
Source: Cancer Med. 2020 Jul 21;9(17):6281–95. doi: 10.1002/cam4.3284 (PMC7476842; doi:10.1002/cam4.3284)
Supplement: Supplementary file 1 — Supplementary Material [file CAM4-9-6281-s001.docx]

***Legends of Supplementary Figures***

******

**Figure S1.**

**Flowchart of datasets screening.** Data were downloaded from the GEO database and filtered based on given criteria.

**Figure S2.**

**Removal of batch effect.** Principal component analysis of 6 independent microarray datasets after correcting for batch effect.

**Figure S3.**

**Validation of RPS and TNBC-RPS in training dataset. A,** RPS is higher in pCR than RD samples in training dataset. The statistical significance is calculated by Wilcoxon rank sum test. **B,** Receiver Operating Characteristic (ROC) curves for pCR prediction using RPS as feature. **C,** TNBC-RPS is higher in pCR than RD samples in TNBC patients in training dataset. The statistical significance is calculated by Wilcoxon rank sum test. **D,** Receiver Operating Characteristic (ROC) curves for pCR prediction in TNBC patients in training dataset using TNBC-RPS as feature. **E,** StromalScore is lower in pCR than RD samples in TNBC patients in metadata. The statistical significance is calculated by Wilcoxon rank sum test. **F,** Receiver Operating Characteristic (ROC) curves for pCR prediction in TNBC patients in training dataset using StromalScore as feature.
